# Adrenal Injury in Situs Inversus

**DOI:** 10.31662/jmaj.2022-0128

**Published:** 2023-03-06

**Authors:** Yuichiro Haba, Naoyuki Hashiguchi

**Affiliations:** 1 Department of General Medicine, Juntendo University School of Medicine, Tokyo, Japan; 2 Department of Emergency and Disaster Medicine, Juntendo University School of Medicine, Tokyo, Japan

**Keywords:** Blunt adrenal injury, Situs inversus, Primary care

A 43-year-old man presented to our primary care clinic with back pain and contusions following a motorcycle accident. Contrast-enhanced computed tomography revealed complete situs inversus, left upper rib fractures, left adrenal enlargement, and no evidence of extravasation or other organ damage. The left adrenal gland measured 3.1 cm along the major axis (Figure 1: arrow) and periadrenal fat stranding (Figure 1: arrowheads) was observed. Serum hormone tests ruled out neoplasia and the enlargement was attributed to trauma ^(1)^. Blunt trauma predominantly affects the right adrenal gland ^(1), (2), (3)^ owing to its proximity to vertebrae and drainage through the inferior vena cava ^(1), (4)^; the patient’s left-sided injury could be attributed to situs inversus. As with other reports ^(2), (3), (4)^, this case was nonoperative and successfully managed in the primary care setting. Our incidental discovery of situs inversus highlights one potentially disastrous consequence of estimating injury severity based on conventional anatomical features and eschewing diagnostic imaging.

**Figure 1.**
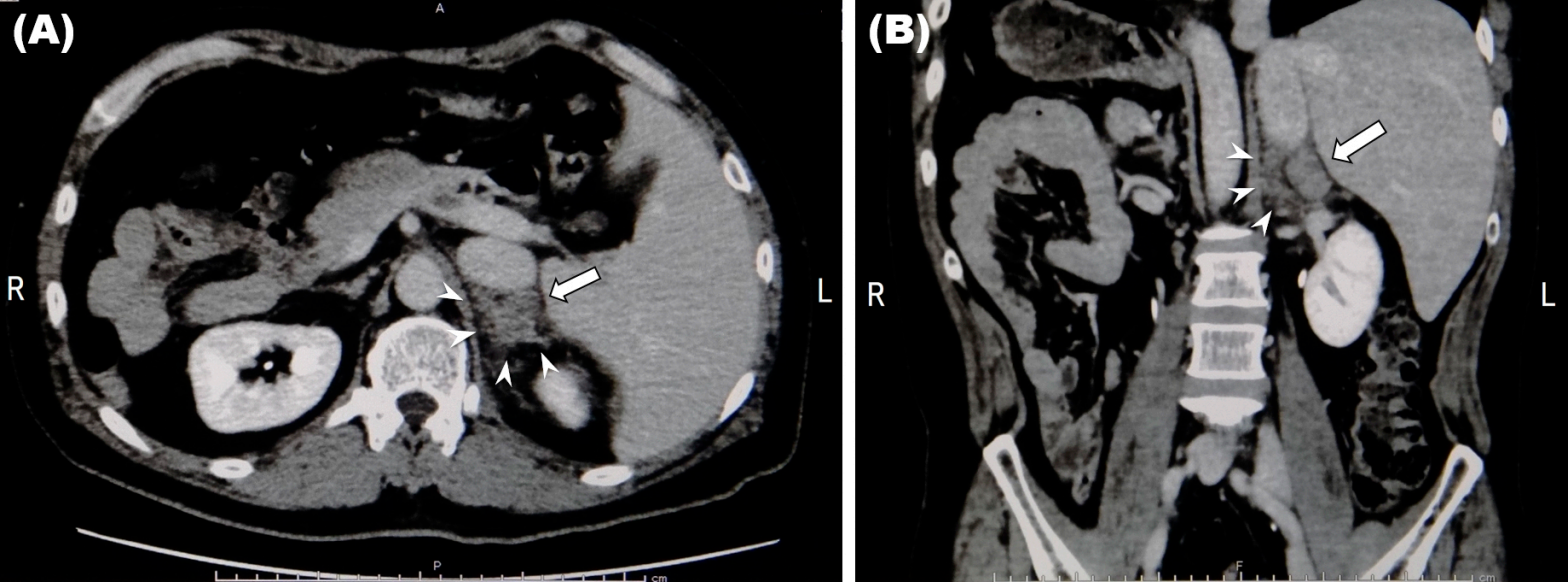
Contrast-enhanced computed tomography showing an enlarged adrenal gland with a 3.1-cm major axis (arrows) and stranding of the periadrenal fat (arrowheads). Other organs are intact. (A): Transverse section. (B): Coronal section.

## Article Information

### Conflicts of Interest

None

### Author Contributions

All authors contributed to patient care. YH conceived the idea for the study and wrote the manuscript. NH contributed to the editing of the manuscript.

### Approval by Institutional Review Board (IRB)

An IRB approval is not required because this is a case report.

### Informed Consent

Written informed consent was obtained from the patient for publication.
